# New Discoveries and Ambiguities of Nrf2 and ATF3 Signaling in Environmental Arsenic-Induced Carcinogenesis

**DOI:** 10.3390/antiox11010077

**Published:** 2021-12-29

**Authors:** Zhuoyue Bi, Yao Fu, Priya Wadgaonkar, Yiran Qiu, Bandar Almutairy, Wenxuan Zhang, Akimasa Seno, Chitra Thakur, Fei Chen

**Affiliations:** 1 Stony Brook Cancer Center, Renaissance School of Medicine, Stony Brook University, Lauterbur Drive, Brookhaven, NY 11794, USA; Zhuoyue.Bi@stonybrookmedicine.edu (Z.B.); Yao.Fu.1@stonybrook.edu (Y.F.); Yiran.Qiu@stonybrookmedicine.edu (Y.Q.); Wenxuan.Zhang@stonybrook.edu (W.Z.); chitra.thakur@stonybrookmedicine.edu (C.T.); 2 Department of Pharmaceutical Sciences, Eugene Applebaum College of Pharmacy and Health Sciences, Wayne State University, 259 Mack Avenue, Detroit, MI 48201, USA; Priya.wadgaonkar@wayne.edu (P.W.); Almutairy@wayne.edu (B.A.); aseno@okayama-u.ac.jp (A.S.); 3 Department of Pathology, Renaissance School of Medicine, Stony Brook University, 101 Nicolls Road, Brookhaven, NY 11794, USA

**Keywords:** arsenic, Nrf2, carcinogenesis, ATF3, ChIP-seq

## Abstract

Environment exposure to arsenic had been linked to increased incidents of human cancers. In cellular and animal experimental systems, arsenic has been shown to be highly capable of activating several signaling pathways that play critical roles in cell growth regulation, malignant transformation and the stemness of cancer stem-like cells. Emerging evidence indicates certain oncogenic properties of the Nrf2 transcription factor that can be activated by arsenic and many other environmental hazards. In human bronchial epithelial cells, our most recent data suggested that arsenic-activated Nrf2 signaling fosters metabolic reprogramming of the cells through shifting mitochondrial TCA cycle to cytosolic glycolysis, and some of the metabolites in glycolysis shunt the hexosamine biosynthesis and serine-glycine pathways important for the energy metabolism of the cancer cells. In the current report, we further demonstrated direct regulation of oncogenic signals by arsenic-activated Nrf2 and connection of Nrf2 with ATF3 stress transcription factor. Meanwhile, we also highlighted some unanswered questions on the molecular characteristics of the Nrf2 protein, which warrants further collaborative efforts among scientists for understanding the important role of Nrf2 in human cancers either associated or not to environmental arsenic exposure.

## 1. Introduction

Globally, there are about 94 million to 220 million people in more than 70 countries facing environmental exposure to arsenic [1]. As one of the most abundant metalloids in Earth’s crust, arsenic is found in high concentrations in some special geographic settings, such as sedimentary rock, ground water, volcanic ashes, metal ores, coal, soil, etc. [2]. In addition, some industry activities, including mining, metal refining, wood preservation, drug development, and manufacture of pesticides, can produce high concentration of arsenic in the working environment and cause occupational exposure to arsenic [3]. The most common environmental arsenic exposure is from drinking water contamination due to the use of source water containing high amounts of arsenic leached from natural rock or soil, in which the arsenic level is much higher than the provisional guideline value of 10 ppb established by both World Health Organization (WHO) and United States Environmental Protection Agency (EPA).

Despite many questions remaining to be fully answered on how environmental exposure to arsenic, especially for inorganic trivalent arsenic, is carcinogenic to humans, arsenic is the first suspected human carcinogen in history. In Paracelsus’s observational study on metal miners’ “mala metallorum” in the 16th century, he believed that sustained exposure to arsenic-containing dust and air in mining and processing of natural ores is responsible for pulmonary symptoms, (most likely lung cancer), and cachexia of the miners [4,5]. A large body of epidemiologic studies had indicated a significant dose–response relationship between arsenic concentration in drinking water and incidences of lung cancer and other malignancies in both men and women [6]. This notion was further supported by direct evidence showing medicinal use of Fowler’s solution (1% potassium arsenite, KAsO_2_) and the development of lung cancer in patients with psoriasis, anemia, or rheumatic diseases [7]. Accordingly, both the International Agency for Research on Cancer (IARC) and US EPA had classified arsenic as Group 1 and Group A carcinogen known to humans, respectively. Although it is mechanistically unclear how ingestion of arsenic from drinking water or arsenic-contaminated food contributes to lung cell malignancy, several epidemiological studies and meta-analysis unraveled strong association of arsenic exposure and impairment of lung function, such as the inverse relationship between arsenic exposure and both FEV1 and FVC, suggesting that absorbed arsenic is able to reach to the lung tissue and perturb the function of the lung cells [8]. It has been well-documented that arsenic activates several kinases and transcription factors in many types of the cells, such as JNK, PI3K/Akt, STAT3, NF-κB, AP-1, etc. that are major contributors to the malignant transformation of the normal cells [3]. Unlike some classical chemical carcinogens, there is no conclusive evidence that arsenic causes point mutations of the genome. The genotoxicity or chromosomal instability after arsenic exposure is believed largely to be due to the inhibition of DNA repair machinery by arsenic [9].

Nuclear factor erythroid-derived 2-like 2 (NFE2L2), also named nuclear factor erythroid 2-related factor 2 (Nrf2), is a basic leucine zipper (bZIP) transcription factor that serves as a master regulator for the expression of genes involved in redox regulation. Tissue injury, inflammation, stress, and/or some carcinogenic signals can activate Nrf2 through the canonical or non-canonical signaling pathway. In the canonical pathway, extracellular stimuli promote oxidation of the conserved cysteine residues in Kelch-like ECH-associated protein 1 (Keap1), leading to dissociation of Keap1 and Cullin 3 ubiquitin E3 ligase from Nrf2, followed by stabilization of Nrf2 that binds to the antioxidant response element (ARE) on target genes for transcriptional regulation. In the non-canonical pathway, some signals induce sequestration of Keap1 from Nrf2 by SQSTM1 (p62), p21, dieptidyl peptidase III (DPP3), Wilms tumor gene X (WTX), and others, to prevent Nrf2 ubiquitination and proteosomal degradation [10]. However, it is also possible that under certain circumstances, Nrf2 can be activated through both canonical and non-canonical mechanisms.

Arsenic had been shown to be able to activate Nrf2 in several types of cells. In our most recent study, we showed that arsenic activates Nrf2 in bronchial epithelial cell line BEAS-2B cells through a unique mechanism. There is a clear time-dependent activation of Nrf2 accompanied Keap1 degradation induced by arsenic [11]. Inhibition of JNK prevented Nrf2 activation. Meanwhile, we provided evidence showing the importance of Nrf2 and its downstream target HIF1α in arsenic-induced metabolic shift from mitochondrial TCA cycle to glycolysis and the generation of the cancer stem-like cells, which supports the oncogenic role of Nrf2 in cancer development [10]. In our most recent studies, we further discovered direct regulation of Nrf2 on several growth factors critical for cancer cell growth and proliferation, Nrf2 dependent ATF3 expression, and possible different or uncharacterized Nrf2 isoforms.

## 2. Nrf2 Is an Oncogenic Transcription Factor

Since the first discovery of Nrf2 and its regulation on genes mainly in the antioxidant pathways that prevent excessive cellular damages caused by oxidative stress, xenobiotics and some metabolic products, Nrf2 was viewed as a tumor suppressive transcription factor. An attempt to use agents that activate or booster Nrf2 activation was made by some scientists as a measure of cancer chemoprevention [12]. The tumor suppressive-like activity of Nrf2 was supported by observations showing that Nrf2 gene knockout mice exhibited an enhanced susceptibility to carcinogenesis induced by certain chemical carcinogen [13], and Nrf2 overactivation by Keap1 knockout impeded cancer cell metastasis [14].

However, this tumor suppressor-like property of Nrf2 was not fully supported by notions that many human cancers showed an overactivation of Nrf2 resulted from gain-of-functional mutation of the Nrf2 genes, including cancers of esophagus, lung, larynx, skin, etc. [12,15]. In mouse lung cancer model with active mutation of oncogene Kras, Tao et al. [16] found that activation of Nrf2 prevents initiation of chemical carcinogenesis but promotes progression of pre-existing tumors. Meanwhile, others also demonstrated that activation of Nrf2 facilitates tumor progression, metastasis, therapeutic resistance, and confers poor prognosis of the cancer patients [10]. Furthermore, the antioxidant activity of the protein products of the Nrf2-regulated genes can in fact enhance the self-renewal and tumorigenicity of the cancer stem cells through lowing the levels of reactive oxygen species (ROS) that induce differentiation of these cells [17].

The oncogenic role of Nrf2 was also confirmed in our studies of arsenic-induced carcinogenesis [11]. In ChIP-seq experiment, we found that arsenic enriches Nrf2 binding to the genes not only in the known antioxidant pathway but in the glycolytic and oncogenic pathways also. It had been known that many cancer cells and cancer stem cells prefer glycolysis rather than mitochondrial TCA cycle for fast energy generation and consumption. In response to arsenic, Nrf2 nearly regulates every step of the enzymatic reaction of glycolysis, either alone or in combination with HIF1α. The arsenic-induced non-hypoxic expression of HIF1α appears to be Nrf2 dependent. There is a conserved Nrf2 binding element at 32.75 kb upstream of the HIF1 gene. Thus, there is a positive forward loop among Nrf2, HIF1α and glycolytic metabolism in the cellular response to arsenic. In addition to HIF1α that is a known oncogenic factor regulating angiogenesis of the tumor tissue and the hypoxic growth of the cancer cells, a number of other well-documented oncogenes and stemness genes for cancer stem cells, including MYC, SOX2, KLF4, TCF19, NAMPT, BACH1, ZEB1, CD44, EGFR, etc., also showed an enhanced enrichment of Nrf2 binding to the Nrf2 binding elements either in upstream, downstream or promoter region of these genes in response to arsenic treatment. Knockout of Nrf2 through CRISPR-Cas9 gene editing does not affect the basal expression, but significantly prevented inducible expression of these genes in response to arsenic.

**Figure 1:**
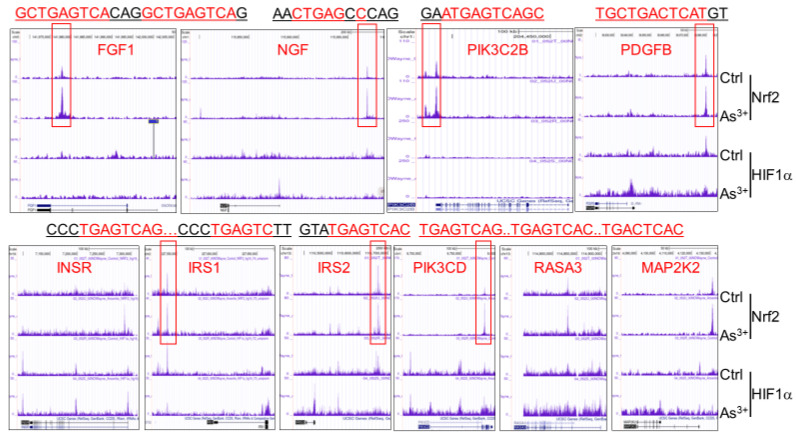
Arsenic-induced expression of growth factors is Nrf2 dependent. Panels show screenshot of Genome Browser for these indicated growth factor genes from ChIP-seq. The major Nrf2 peaks detected on these genes are highlighted with red boxes. The sequences for the conserved Nrf2-binding elements in these indicated Nrf2 peaks are shown on the top of each panel. Data are derived from global ChIP-seq using antibodies against Nrf2 and HIF1α, respectively, and the control cells and cells treated with 1 μM arsenic (As^3+^) for 6 h.

The contribution of Nrf2 to cancer development might be multifaceted. To expand above observations, we recently investigated possible function of Nrf2 on the transcriptional regulation of several growth factors and found that many of these genes have known Nrf2 binding motif with core sequence TGAGTC or TGACTC, and arsenic treatment enhanced Nrf2 binding to such motifs. These growth factors include FGF1, NGF, PK3C2B, PDGFB, PDGFD, IGF1, NGF, and TGFA (upper panels in Figure 1 and data not shown). The Nrf2 enrichment peaks are located at the upstream of genes for IGF1, PDGFB and PDGFD, downstream of NGF, promoter region of PIK3C2B, and gene body of FGF1 and TGFA. Through visual inspection, Nrf2 binding motifs were identified in all these prominent Nrf2 peaks, except the peak in TGFA gene.

Insulin and its associated downstream signaling pathway are not only master regulators for systemic metabolism, but also serve as growth factors for the growth and proliferation of the cells, such as hepatocytes, fibroblast cells, epithelial cells, and tumor cells [18]. Many human cancers showed elevation of the insulin signaling [19]. The receptors for insulin and insulin-like growth factor (IGF-1) are ubiquitously expressed in cancer cells. Thus, either paracrine or autocrine insulin signaling can provide growth advantages or antiapoptotic potentials for the cancer cells. Although the insulin signaling was not top-ranked in the pathway analyses of the Nrf2-regulated genes in the cells treated with arsenic, majority of the genes in this pathway showed a strong arsenic-induced enrichment of Nrf2 binding in ChIP-seq experiment, including insulin receptor (INSR), insulin receptor substrate 1 (IRS1), IRS2, PIK3CD (PI3K), RASA3, MAP2K2 (MEK2) (bottom panels in Figure 1). The conserved Nrf2 binding elements were found in the Nrf2 binding peak regions in the genes of IRS1, IRS2 and PIK3CD. Interestingly, three consecutive Nrf2 binding elements were identified in the 9th intron region of the PIK3CD gene (Figure 1). RNA-seq data revealed that deletion of Nrf2 gene significantly reduced the expression of PIK3CD (data not shown). All these findings unequivocally suggest that Nrf2 is a pivotal regulator for the insulin signaling important for carcinogenesis and tumorigenesis.

**Figure 2:**
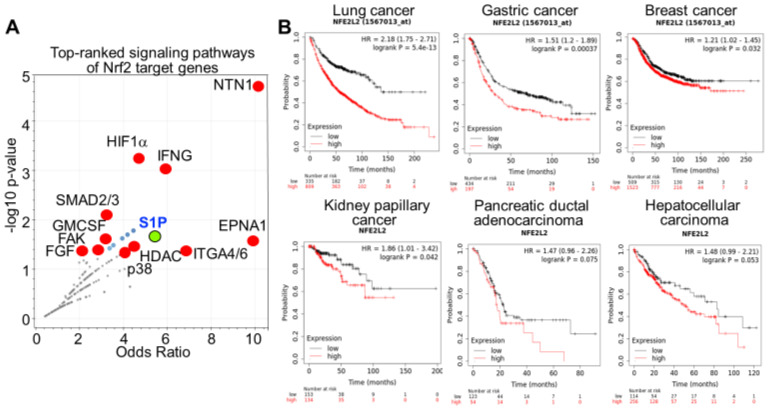
Nrf2 is an oncogenic transcription factor. (**A**). Signaling pathway assay for those genes that showed an enhanced Nrf2-binding in ChIP-seq in the cells treated with 1 μM arsenic for 6 h. (**B**). Kaplan–Meier survival analysis of the patients with the indicated cancers stratified by the low and high expression of the Nrf2 gene (NFE2L2). Top panels for lung cancer, gastric cancer and breast cancer were derived from Kaplan-Meier Plotter mRNA gene chip database, and bottom three panels for cancers in kidney, pancreas and liver were derived from Kaplan–Meier Plotter RNA-seq data sets. The statistical significance in patient survival was determined by log rank test and Cox’s proportional hazards model.

An additional “NCI-Nature 2016” pathway assay using Enrichr program for 663 arsenic-enhanced Nrf2 target genes revealed several additional growth regulation pathways that are top-ranked, such as signaling pathways of HIF1α, SMAD2/3, S1P, GMCSF, FAK, and FGF (Figure 2A). In addition to promote vascularization of the tumor mass, HIF1α had also been linked to the generation of CSCs through upregulation of glycolysis and the expression of a number of stemness genes [11]. Although there is no information directly linking SMAD2/3 to CSCs, the downstream effector of TGFβ signaling, SMAD2/3, is highly capable of maintaining an important pluripotent transcriptional network in naïve human pluripotent stem cells, including the expression of stemness genes, such as Nanog, KLF4, CDK19, etc. [20]. It is known that both CSCs and normal stem cells share similar pluripotent circuits for self-renewal and differentiation. Thus, overexpression of SMAD2/3 by the arsenic-induced Nrf2 must play critical role in the generation of CSCs. The growth factor signaling pathways of GMCSF, FAK and FGF may play auxiliary role on the reprogramming and overall growth potential of the cancer cells and CSCs. It is worth noting that the S1P (sphingosine-1-phosphate) pathway is highlighted in this analysis for the Nrf2 target genes. This pathway was also enriched in our ChIP-seq analysis for the genes that acquired chromatin status featured with active histone trimethylation marker, H3K4me3, and reduced level of repressive trimethylation marker, H3K9me3 and/or H3K27me3, in the arsenic-induced transformed cells and cancer stem cells [21]. As a versatile lipid metabolite, S1P has been implicated in the regulation of immune response, inflammation and angiogenesis [22]. Through either receptor dependent or non-receptor dependent mechanisms, S1P has been shown to be able to promote V12Ras-mediated transformation, the growth and survival of the cancer cells. Several types of human tumors manifested elevated expression of S1P receptors [23]. Using ALDH1+ population of the breast cancer cells, studies by Hirata et al. [24] suggested that S1P produced by SPHK1 interacts with the cell surface receptor, S1PR3, followed by inducing p38-dependent phosphorylation and activation of metallopeptidase, ADAM17, which cleaves the cytosolic domain of Notch receptor, leading to ligand-independent activation of Notch signaling and the expansion of the cancer stem cell population. The notion of oncogenic property of Nrf2 is further supported by the correlation of higher Nrf2 gene expression and poorer survival of cancer patients with lung cancer, gastric cancer, breast cancer, kidney papillary cancer, and likely pancreatic adenocarcinoma and hepatocellular carcinoma, although the latter two have a moderate statistics value due to the relatively small sample size (Figure 2B).

**Figure 3:**
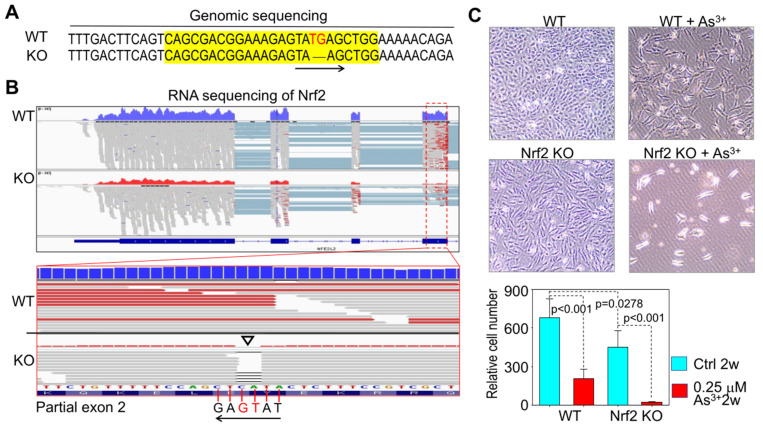
Gene knockout of Nrf2 prevents arsenic-induced malignant transformation. (**A**). BEAS-2B cells were subjected to CRISPR-Cas9 gene editing using sgRNA that targets the exon 2 region of the Nrf2 genes. Successful knockout of Nrf2 gene was confirmed by genomic sequencing. The sequences highlighted with yellow color are the sgRNA targeting region in CRISPR-Cas9 editing. The knockout (KO) cells showed “thymine-guanine (TG)” deletion. (**B**). RNA-seq showed deletion of the TG complement nucleotides cytosine-adenine (CA) in the exon 2 transcripts in Nrf2 KO cells (pointed by an open triangle). (**C**). Cell viability analysis of the WT and Nrf2 KO cells in the absence or presence of 0.25 μM arsenic (As^3+^) for 20 days. Cell numbers were averages of cell counting in four randomly selected microscopic fields. Sigma plot t test was used to determine the statistical significance. *p* < 0.05 was considered statistically significant.

Since environmental arsenic exposure is a long-term process, and cancer development usually takes place after years to decades. The question to be answered is: does Nrf2 play an essential role in the malignant transformation of the noncancerous cells treated with environmentally relevant concentrations of arsenic consecutive to what we performed previously [11]? To address this question, we established Nrf2 knockout (KO) cell lines through CRISPR-Cas9 gene editing using the human bronchial epithelial cell line, BEAS-2B. Genomic sequencing revealed a deletion of TG in exon 2 of Nrf2 gene in the KO cells (Figure 3A). This genomic deletion was validated additionally by RNA-sequencing (RNA-seq), which showed not only an overall reduced expression of Nrf2 mRNA in the KO cells, but a two-nucleotide deletion corresponding to the TG deletion in the genome (Figure 3B). Next, we tried to induce cell transformation using the wild-type (WT) and Nrf2 KO cells by consecutive treatment of these cells with 0.25 μM arsenic for three to six months, the same procedure we employed before. During the first week of this experiment, there is no significant difference in cell proliferation between WT and Nrf2 KO cells with or without arsenic treatment. After 20 days of cell passaging, however, more than 90% of the KO cells treated with arsenic died (Figure 3C). Under control condition, a slower proliferation of the KO cells relative to the WT cells was noted. Accordingly, although the transformation experiment was aborted at 20 days due to arsenic-induced massive cell death of the KO cells, we concluded that Nrf2 is essential for arsenic-induced transformation, which again strengthened the conclusion that Nrf2 is an oncogenic transcription factor.

## 3. Nrf2 Dependency of Arsenic-Induced Expression of ATF3, a Stress Response Oncogene

Activating transcription factor 3 (ATF3) is a stress-inducible AP-1/ATF/CREB family member involved in metabolism, inflammation, innate immunity, and carcinogenesis [25]. It has been demonstrated that homodimer of ATF3 may serve as a repressor for some of its target genes, whereas heterodimer of ATF3 with c-Jun is most likely an activator for the transcription of its target genes. In addition, the transcriptional activity of ATF3 is also depending on the types of ATF3 variants. The full-length isoform of ATF3 may be repressive. In contrast, the shorter isoform that lacks the leucine zipper motif resulted from alternative splicing of pre-mRNA is capable of sequestering inhibitory co-factors and promotes transcription. The evidence of cancer promoting of ATF3 was provided by studying keratinocyte-derived squamous cell carcinoma, which showed that increased ATF3 expression accounts for suppression of p53-dependent senescence and enhanced tumorigenic potential [26]. This notion was supported by an upregulation of ATF3 expression in human breast cancer [27], prostate cancer, Hodgkin’s lymphoma [28,29], lung cancer [30], and spontaneous multifocal keratinocyte tumors in mice carrying a mesenchymal-specific deletion of CSL/RBP-Jκ [31].

The regulatory role of ATF3 on metabolism was considered as a key driving factor for the aggressiveness of acute myeloid leukemia (AML) [32]. ATF3 has properties in advancing cell cycling and preventing differentiation, most likely through its transcriptional regulation on serine synthesis and one-carbon metabolism that maintain purine and pyrimidine pools in the AML cells. This effect of ATF3 resembles the metabolic features in the arsenic-induced cancer stem-like cells that showed an enhanced glycolytic metabolism and the shunting of glycolytic metabolites into the serine/glycine pathway for one-carbon metabolism [11]. Importantly, most of the ATF3-regulated genes encoding enzymes in the serine/glycine pathway are also target genes of arsenic-induced Nrf2 [11]. In human melanoma cells and non-small cell lung cancer cells, ATF3 binds to the promoter region of PD-L1 gene to foster expression of PD-L1, leading to evasion of the cancer cells from T cell-mediated cell-killing [30]. Even in the noncancer host cells, expression of ATF3 dispenses a pro-metastatic microenvironment in the lung for chemotherapy-induced metastasis of the primary breast cancer cells [33]. In wild-type mice inoculated with MVT-1 breast cancer cells at the orthotopic site, the fat pad, administration of the chemotherapeutic drug paclitaxel (PTX) reduced the size of primary tumors, but exacerbated lung metastasis of the tumor cells. In ATF3 knockout mice, however, such metastasis was abolished almost completely. A detailed biochemical analysis unraveled that ATF3-dependent expression of pro-angiogenic genes, including ANGPT1, Notch1, CX3CL1, KDR (VEGFR2), but not the anti-angiogenic genes, such as TNFα, CXCL9, CXCL10, CXCL11, CXCL14, is responsible for the migration of the tumor cells from the primary sites. Meanwhile, ATF3 is also important for the colonization of the cancer cells in the metastatic sites in the lung through prompting expression of chemokine C-C motif ligand 2 (CCL2) that recruits inflammatory monocytes and VEGFR1^+^ macrophages that favor metastasis and immunosuppression.

It is currently unknown whether ATF3 contributes to the generation or specialization of the cancer stem cells in human cancers nor the maintenance or self-renewal of the normal stem cells. In hematopoietic stem cells (HSC), transcriptomic and computational analyses suggest that ATF3 is one of the transcription factors regulating transcription of RUNX1, a hematopoietic transcription factor, at the specific ontogeny stages of HSC [34]. In human transformed breast cell line MCF10CA1a, an earlier study by Hai and colleagues [35] revealed ATF3-dependent transcription of several genes in epithelial–mesenchymal transition (EMT), including TWIST1, FN1, SERPINE1, PLAU, CAV1, Snail (SNAI1) and Slug (SNAI2). Since some cancer stem cells acquired EMT potentials, the connection between ATF3 and EMT may indicate an important perspective of ATF3 in the functional specialization of the cancer stem cells. This assumption is well-corroborated to the finding that transgenic overexpression of ATF3 in mouse basal epithelium of the mammary gland causes mammary carcinomas with predominant activation of the Wnt/β-catenin pathway [36], a pathway involved in the stemness of many cancer stem cells found in different types of experimental or human cancers [37].

**Figure 4:**
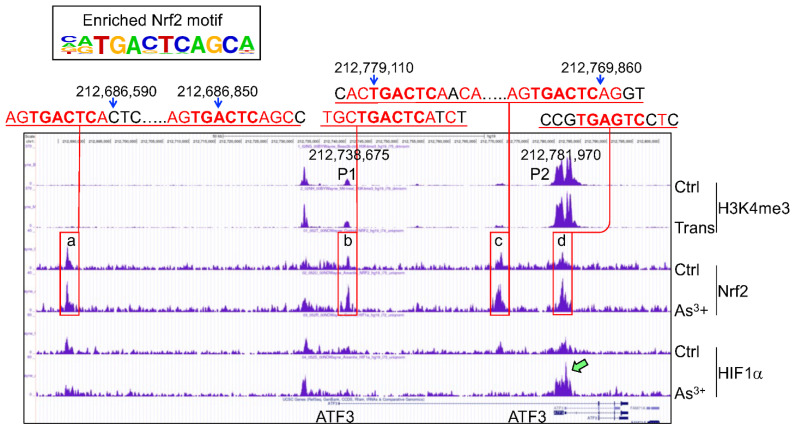
Nrf2-dependent expression of ATF3. Panel shows screenshot of H3K4me3 ChIP-seq of the control cells and transformed cells (Trans), and Nrf2 and HIF1α ChIP-seq of the control cells and the cells treated with 1 μM arsenic (As^3+^) for 6 h, for the ATF3 gene. P1 and P2 represent the distal and proximal ATF3 promoters, respectively. Red boxes indicate the major Nrf2 peaks in the ATF3 gene. The conserved Nrf2 elements are shown on the top of each box. Numbers on the top of Nrf2 elements indicate the relative positions of these elements in human genome (hg19). Top left box shows the enriched Nrf2 binding motif as determined by the HOMER program in ChIP-seq.

Overwhelming evidence suggests that neither mRNA nor protein of ATF3 is barely detectable in normal tissues or cells under physiological condition [35]. In response to DNA damage, hypoxia, chemical carcinogen, or chemotherapeutic drugs, several transcription factors, including Nrf2, MYC, E2F, AP1, ATF/CREB, NF-κB, HIF1α, p53, and others [38,39], had been linked to the stress-induced expression of ATF3 in different types of the cells. In human and mouse primary brain astrocytes, several lines of evidence indicated a Nrf2 dependency of ATF3 expression [38]. First, classic Nrf2 activators, tBHQ and BHA, induced both mRNA and protein of ATF3. Second, genetic deficiency of Nrf2 gene in mouse embryonic fibroblasts prevented ATF3 induction. Third, deletion of the putative antioxidant response element in ATF3 promoter blocked the Nrf2-dependent luciferase activity. Lastly, chromatin immunoprecipitation (ChIP) analysis revealed Nrf2 binding to the ARE element of the ATF3 promoter. In our recent ChIP-seq assay of the control BEAS-2B cells and the BEAS-2B cells treated with 1 μM arsenic for 6 h, we identified at least four Nrf2 binding peaks in P1 and P2 promoters, intron, and upstream of the ATF3 gene (Figure 4). Further inspection unraveled one or two Nrf2 binding ARE elements in each of these peaks. Arsenic treatment enhanced the enrichment of Nrf2 binding to all of these ARE elements.

**Figure 5:**
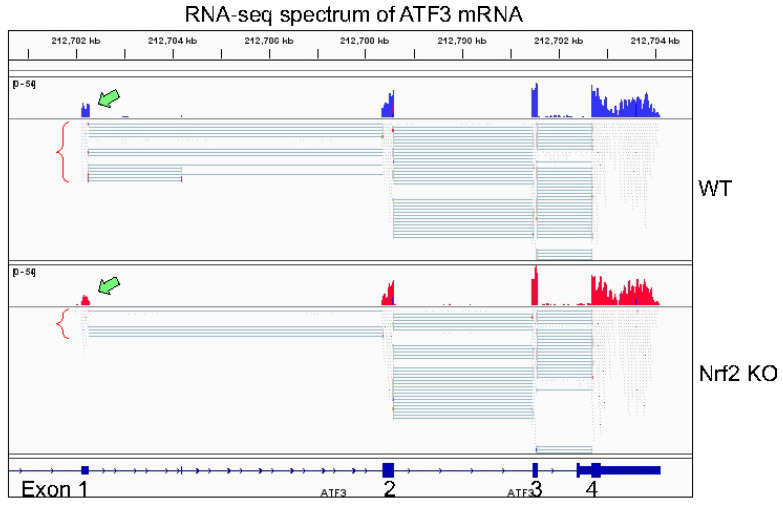
Knockout of Nrf2 reduced ATF3 expression. RNA-seq spectrums were shown for the WT and Nrf2 KO cells. The readout of exon1 of ATF3 is pointed by green block arrows.

The human ATF3 gene is located in chromosome 1q32.3 region. Due to the use of alternative exon1 and promoters, and alternative splicing sites in the exon-intron conjuncture, several ATF3 transcript variants had been identified [39,40]. Depending on the types of stimuli, the usage of promoter 1 (P1) or P2 may be different. The P1 promoter is located 43,295 bp upstream of the P2 promoter and is more active in response to serum stimulation [41] (Figure 4). In the arsenic-transformed human bronchial epithelial cell line BEAS-2B cells, however, we found the P2, but not P1, is much active, as evidenced that the degree of H3K4me3 enrichment in P2 promoter is much higher than the P1 promoter (Figure 4). RNA-seq failed to detect transcripts derived from P1 promoter, but only the transcripts in corresponding to P2 promoter in both wild-type and Nrf2 KO cells (Figure 5). Among the transcriptional variants of ATF3, two variants are particularly interesting. The first one translates ATF3 proteins with a C-terminal 118 amino acids truncation, leading to the deletion of leucine-zipper region (ATF3Dzip), and the second one translates a protein with C-terminal shift of open-reading-frame (ORF) for 135 amino acids (ATF3Dzip2). The leucine-zipper region is essential for dimerization of ATF3 protein with other transcriptional partners. Certain stress signals favor the production of ATF3Dzip2 in the cells [42]. In pancreatic cancer cells, studies by Kha et al. [41] suggested that TGFβ induces ATF3, most likely through activating the distal P1 promoter, whereas activation of Nrf2 preferentially induces ATF3Dzip2 through the using of the proximal P2 promoter. More remarkably, analysis of cell apoptosis and tumorigenesis in mice revealed that ATF3 has proapoptotic effect on cancer cells and has limited effect on tumor growth. In contrast, the Nrf2-dependent ATF3Dzip2 is antiapoptotic and tumor growth promoting, which further supports the oncogenic effect of Nrf2 as discussed earlier.

**Figure 6:**
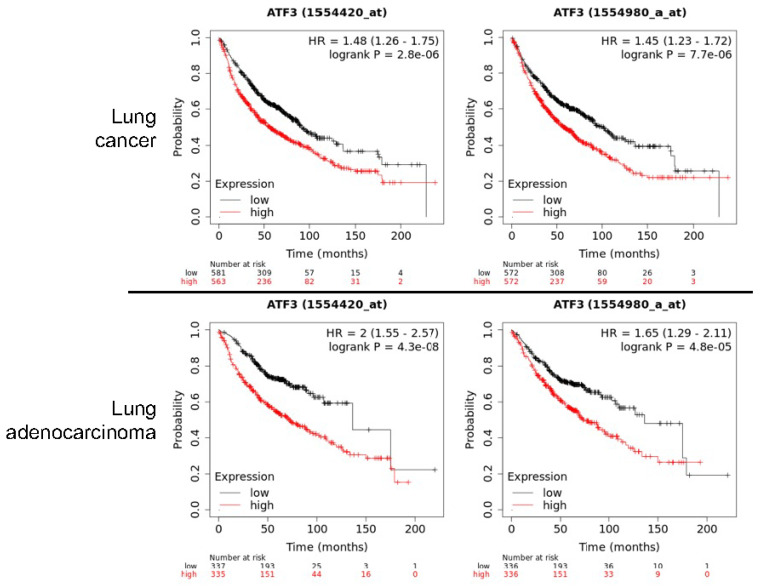
Increased expression of ATF3 predicates poorer survival of the lung cancer patients. Data are derived from Kaplan–Meier Plotter survival analysis of the total lung cancer patients (**top**) and the lung adenocarcinoma (**bottom**). Two different ATF3 gene probes were included in this analysis.

Our RNA-seq experiment is partially consistent with the findings described above. In both WT cells and Nrf2 KO cells, only transcripts derived from P2 promoter of ATF3, the proximal promoter, were detected (Figure 5). Knockout of Nrf2 resulted in a significant decrease of transcripts from P2 promoter and the readout of exon1 in RNA-seq (pointed by green block arrow in Figure 5). Although it is arbitrary and speculative, we believe that the following reasons may explain such a unique pattern of ATF3 transcription. First, relative to P1 promoter, the P2 promoter of ATF3 is more active based on the enrichment level of H3K4me3, an active promoter marker, in ChIP-seq (Figure 4). Second, the levels of basal and arsenic-induced Nrf2 binding in P2 promoter (box d in Figure 4) is much stronger then in P1 promoter (box b). Third, there is an additional Nrf2 binding peak (box c) that has two conserved and several other potential Nrf2 binding elements, which is 12 kb upstream of P2 promoter. This Nrf2 peak may facilitate the activation of P2 promoter. Lastly, there is a clear arsenic-enhanced HIF1α binding peak at the P2 promoter (pointed by green block arrow in Figure 4), which is not presented in the P1 promoter. The Nrf2 peak (box a), located 51.8 kb upstream of P1 promoter (box b), may also play role on the arsenic-induced ATF3 expression. There is another Nrf2 binding peak 27.1 kb upstream of box a (not shown in Figure 4), which contains two conserved Nrf2 binding elements, CTCTGACTCCCT (position 212,659,230) and GGGTGACTCAGCG (position 212,659,450). Because of these multiple conserved Nrf2 binding elements, these observations, thus, unequivocally suggest that Nrf2, especially in the condition of environmental arsenic exposure, is a central transcription factor that mediates the oncogenic ATF3 expression. The oncogenic notion of ATF3 is further supported by the poorer survival of the lung cancer patients with higher level of ATF3, especially among the patients with lung adenocarcinoma (Figure 6).

## 4. Some Ambiguities on the Molecular Characteristics of Nrf2 Protein

**Figure 7:**
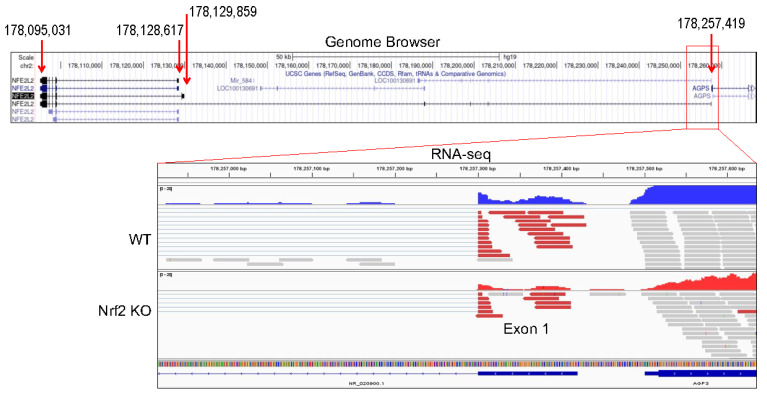
Genomic structure and main transcripts of the Nrf2 gene (NFE2L2). The shared promoter region of the longest transcript of Nrf2 with the AGPS gene is indicated by a red box. Bottom panel shows transcription of exon 1 of the longest Nrf2 transcript in WT and Nrf2 KO cells as determined by RNA-seq.

The human Nrf2 (NFE2L2) gene is mapped on chromosome 2q31.2. The most documented Nrf2 precursor transcripts (pre-mRNAs) span 33,586 bp or 34,828 bp with 5 exons in the genome. In the latest genome assembly, there is a predicted transcript of Nrf2 that spans 162,388 bp on the genome. The tentative transcription start site of this longest Nrf2 transcript is 51 bp upstream of AGPS gene (Figure 7) and contains eight exons. Thus, most likely, this transcript shares promoter with AGPS gene. It is currently unknown whether this longest transcript is truly expressed or not. In our recent RNA-seq analysis for the WT and Nrf2 KO cells, we noted a marginal transcription of the exon 1 of this transcript (bottom panel of Figure 7), indicating a limited degree of expression of this transcript. Since the identification of Nrf2 gene and its encoded Nrf2 protein, a number of antibodies had been developed that can detect Nrf2 proteins with a molecular weight (MW) ranged from 65 to 110 kDa [43]. Based on the amino acid sequence, the predicted MW of Nrf2 is 66 kDa. However, in most of the immunoblotting detection for the human Nrf2 proteins from cells or tissues, the detected MW is around 95 to 120 kDa. Such a discrepancy was tentatively attributed to the abundance of acidic residues found in the Nrf2 protein [44,45].

It has long been known that post-translational modifications, such as phosphorylation, ubiquitination, glycation, etc., and alternative splicing of the pre-mRNA, can change the MW of any given proteins. The first evidence of Nrf2 alternative splicing was provided by Goldstein et al. [46], who revealed splicing skipping of exon 2 or exons 2 and 3 of Nrf2 in some human lung cancer samples and cancer cell lines. The corresponding amino acid sequence of exon 2 is the Neh2 domain that contains two Keap1-interaction motifs DLG and ETGE. Therefore, skipping exon 2 and/or exon3 will generate a gain-of-functional Nrf2 protein that abolishes Keap1 binding and the subsequent Cullin3-E3 ligase-mediated ubiquitination and degradation of the Nrf2 protein by the proteasome. Most recently, studies by Mikac et al. [47] noted a Nrf2 transcript with partial loss of exon 2 resulted from the use of an in-frame splicing site in the 3′-terminus of exon 2 in A549 cells. This alternative splicing also generates a Nrf2 protein lacking Keap1 binding motifs, leading to stabilization of the Nrf2 protein. A question that remains to be answered is how common gain-of-functional Nrf2 alternative splicing occurs in human cancers. It will be also interesting to determine whether these bands with a MW smaller than 90 kDa detected by certain Nrf2 antibodies in some immunoblotting are products of alternative splicing of the Nrf2 pre-mRNA.

The specificity of antibodies that are widely used currently to examine the level of Nrf2 protein in Western blotting is another topic that has been extensively discussed thus far [43]. Lau and colleagues [44] tested anti-Nrf2 antibodies from three different commercial sources and found that one antibody detected a predominant Nrf2 band with a MW of 110 kDa, and concluded that the authentic Nrf2 protein in immunoblotting should be the band at a migrating position around 110 kDa. Another two antibodies detected multiple bands with strong signals at migrating positions of 40 to 100 kDa, in addition to the faint Nrf2 band at position of 110 kDa. Using mouse embryonic fibroblast cells from WT and Nrf2 knockout mice and SC-C20 antibody in immunoblotting, the Nrf2 knockout cells not only showed elimination of the 110 kDa Nrf2 band, but other bands observed in wild-type fibroblast cells with MW from 90 to 180 kDa are also undetected. Similarly, Kemmerer et al. [48] compared anti-Nrf2 antibodies D1Z9C (Cell Signaling Rabbit mAb #12721, recognizing Nrf2 peptides surrounding Ala275) with EP1808Y (Abcam62352, Rabbit mAb, recognizing Nrf2 peptide from amino acid 550 to C-termus), SC-C20 and H300, and found D1Z9C has the highest specificity to Nrf2 protein in immunoblotting. In A549 cells and squamous cell carcinoma RERF-LC-AI cells, both D1Z9C and EP1808Y can detect Nrf2 proteins with MW of 130 and 105 kDa [47]. The 130 kDa Nrf2 appears to be a phosphorylated form of Nrf2 that is sensitive to ubiquitination and degradation mediated by Keap1-Cullin3. The 105 kDa Nrf2 is somehow more stable. It is unclear whether the 105 kDa Nrf2 is a product of the transcript with partial loss of exon 2 as mentioned above.

**Figure 8:**
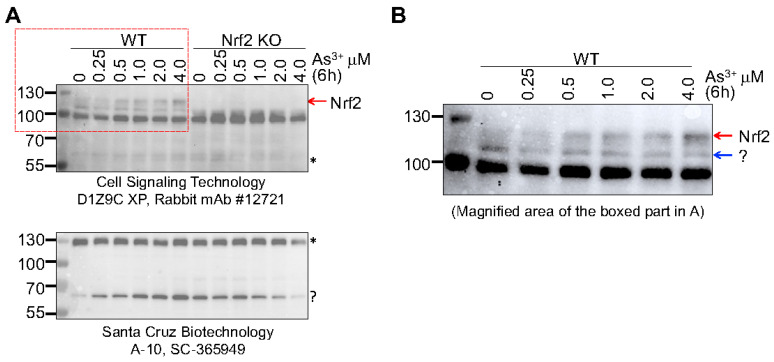
Specificity analysis of the anti-Nrf2 antibodies from different commercial sources. (**A**) The WT and Nrf2 KO cells were treated with the indicated concentrations of arsenic (As^3+^) for 6h followed by immunoblotting using D1Z9C antibody (top) and SC-365949 antibody (bottom), respectively. (The D1Z9C antibody had shown nearly identical specificity with the Abcam62352/EP1808Y antibody). The authentic Nrf2 band is marked by a red arrow. Asterisks and question mark denote the unknown characteristics of these protein bands. (**B**) Magnified potion of the top left panel in A to show the duplex of the Nrf2 band.

We had explored the specificity of two anti-Nrf2 antibodies from different commercial sources for immunoblot detection of Nrf2 using WT and Nrf2 KO BEAS-2B cells [11,43]. The anti-Nrf2 antibody CS-D1Z9C detected two bands at position around 95 kDa and 110 kDa in WT cells. Knockout of Nrf2 removed the 110 kDa band completely, but not the band of 95 kDa, suggesting that the 110 kDa protein band is the true Nrf2 protein. When the mouse monoclonal anti-Nrf2 antibody SC-365949 was used in this immunoblot, 3 protein bands at positions of 130, 70 and 40 kDa were detected. Knockout of Nrf2 had no effect on the bands at 130 and 70 kDa but removed the 40 kDa band [43]. To clarify this further, we recently evaluated these two antibodies using the cells treated with different concentrations of arsenic from 0 to 4 μM for 6 h. There was a clear dose-dependent induction of the 110 kDa Nrf2 band in WT cells in the immunoblot with CS-D1Z9C antibody, which was not detected in the Nrf2 KO cells (Figure 8A). The 95 kDa band was consistent in both WT and KO cells and was not affected by the arsenic treatment. The SC-365949 antibody detected two major protein bands around 130 and 70 kDa, respectively, in both WT and KO cells. Arsenic appears to be able to induce the 70 kDa band in the WT cells. Deletion of Nrf2 did not remove the 70 kDa band but prevented its induction by arsenic, suggesting that this band represented a non Nrf2 protein and its induction by arsenic is Nrf2 dependent (Figure 8, bottom panel). Careful examination of the 110 kDa band detected by the CS-D1Z9C anti-Nrf2 antibody in the WT cells revealed that this band is a duplex, and only the top band (pointed by a red arrow), but not the bottom band (pointed by a blue arrow), can be induced by arsenic (Figure 8B).

**Figure 9:**
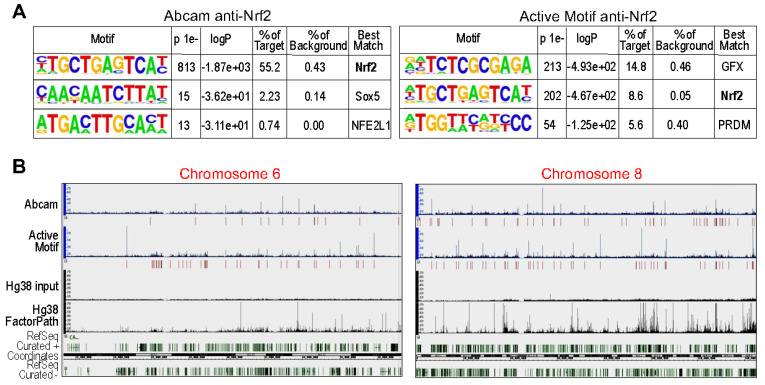
Two anti-Nrf2 antibodies showed different specificity and coverage in ChIP-seq. (**A**) HOMER analysis of the enriched motifs of the ChIP-seq peaks produced by the use of these two different antibodies. (**B**) Limited overlap of the ChIP-seq peaks between the indicated two antibodies in ChIP-seq.

As an additional measure to evaluate the accuracy of anti-Nrf2 antibodies, we also performed a ChIP-seq analysis using antibody EP1808Y (Abcam62352), which showed similar specificity of the CS-D1Z9C as investigated by Mikac et al. [47], and antibody AB_2793695 (Active Motif cat#61599), which detected a 95 kDa protein band as indicated by the datasheet. Two ChIP reactions were carried out using 30 μg of proteins from the arsenic-treated BEAS-2B cells. The ChIP DNAs were processed into standard Illumina ChIP-seq libraries and sequenced to generate more than 5 million reads aligned to the human genome (hg38). After removal of duplicate and non-uniquely mapped reads, about 5.5 and 6 million alignments were obtained for the Abcam and Active Motif antibody assay, respectively. To examine the specificity of the results, peak sequences were searched for enriched motifs using the HOMER program. The results showed that 55.2% of the Abcam peaks matches the Nrf2 binding (ARE) element with a core sequence of TGCTGAGTCA, whereas only 8.6% of the Active Motif peaks matches this Nrf2 binding element (Figure 9A). About 15% of the Active Motif peaks contained an uncharacterized GFX element that is mostly detected in the promoter region of genes, and 6% of these Active Motif peaks had PRDM motif. In addition, the peak locations on the genome of these two ChIP-seq reactions are different significantly, only about 20% to 30% of the peaks from these two analyses overlap to each other, although the Active Motif peaks are relatively stronger than the Abcam peaks (Figure 9B). Taken together, we believe that the antibody EP1808Y, the Abcam antibody, is more accurate in recognizing the Nrf2 protein. In addition to Nrf2, the Active Motif antibody, however, may also have affinities with some promoter-binding proteins.

## 5. Conclusions

More than 20,000 papers on Nrf2 has been published since its first discovery in 1994 by Moi, et al. [45]. The mechanisms of Nrf2 activation and activity in redox regulation had been well-established. Although it is still a topic of debate whether Nrf2 is tumor suppressive or pro-tumorigenic, accumulating evidence suggests that Nrf2 acts more similar to a culprit in oncogene activation, malignant transformation and tumorigenesis. Our studies on arsenic-induced carcinogenesis further confirmed the regulatory role of Nrf2 on oncogenesis, glycolytic metabolism and generation of growth factors that provide a favorite environment for cancer development. Furthermore, the latest ChIP-seq data also linked Nrf2 to the expression of genes in angiogenesis (e.g., ANGPT1, VEGFC, CCL2), maintenance of the stem cells or cancer stem cells (PRDM16, TULP4, RUNX, CTNNB1, ULK4, etc.), antagonization of the p53 signals (MDM2, TP63, etc.), and genes that regulate methylation of the DNA and histone proteins. All these actions ultimately underscore the dark side of Nrf2 that serves as a hallmark of cancer.

The current emphasis on the dark side of Nrf2 is mostly focused on the Nrf2 signaling in the tumor cells, whereas the possible impact of Nrf2 on the behavior of tumor-associated immune cells, macrophages and fibroblast cells is unexplored. Emerging evidence suggests that, in addition to the malignant nature of the cancer cells, many types of tumor-associated cells in the tumor microenvironment are also critical determinants for the tumorigenesis, heterogenicity of the tumors, therapeutic responses, and cancer cell metastasis. We now know that Nrf2 is one of the important regulators for metabolism. We had also learned that the cell lineage development and functional specialization of some tumor-associated T cells, including T helper cell 17 (Th17) and T-regulatory cells (Treg) are largely depending on the metabolic status inside of these cells. Our unpublished data also indicated Nrf2 dependency, at least partially, in the expression of immune checkpoint proteins PD-1 and PD-L1. Thus, activation of Nrf2 as well as its down-stream target ATF3 will influence the cellular and cytokine dynamics in the tumor microenvironment. Accordingly, more effort should be made in future studies to define the roles of Nrf2 signaling on the functions or activities of the non-cancerous cells in the tumor microenvironment during carcinogenesis and tumorigenesis, either associated or not, with environmental arsenic exposure.
